# Linking Adiponectin and Its Receptors to Age-Related Macular Degeneration (AMD)

**DOI:** 10.3390/biomedicines11113044

**Published:** 2023-11-14

**Authors:** Mayank Choubey, Munichandra B. Tirumalasetty, Nalini S. Bora, Puran S. Bora

**Affiliations:** 1 Department of Foundations of Medicine, NYU Grossman Long Island School of Medicine, Mineola, NY 11501, USA; choubeymayank48@gmail.com (M.C.); tmunichandrababu@gmail.com (M.B.T.); 2 Department of Ophthalmology, Jones Eye Institute, Pat & Willard Walker Eye Research Center, University of Arkansas for Medical Sciences, 4301 West Markham, Little Rock, AR 72205, USA; nbora@uams.edu

**Keywords:** obesity, adiponectin (APN), adiponectin receptors (AdipoRs), age-related macular degeneration (AMD), dry AMD, wet AMD, choroidal neovascularization (CNV), angiogenesis, inflammation

## Abstract

In recent years, there has been a captivating focus of interest in elucidating the intricate crosstalk between adiponectin (APN), a versatile fat-associated adipokine and ocular pathologies. Unveiling the intricate relationship between adipocytokine APN and its receptors (AdipoRs) with aging eye disorders has emerged as a fascinating frontier in medical research. This review article delves into this connection, illuminating the hidden influence of APN on retinal health. This comprehensive review critically examines the latest findings and breakthroughs that underscore the pivotal roles of APN/AdipoRs signaling in maintaining ocular homeostasis and protecting against eye ailments. Here, we meticulously explore the intriguing mechanisms by which APN protein influences retinal function and overall visual acuity. Drawing from an extensive array of cutting-edge studies, the article highlights APN’s multifaceted functions, ranging from anti-inflammatory properties and oxidative stress reduction to angiogenic regulation within retinal and macula tissues. The involvement of APN/AdipoRs in mediating these effects opens up novel avenues for potential therapeutic interventions targeting prevalent aging eye conditions. Moreover, this review unravels the interplay between APN signaling pathways and age-related macular degeneration (AMD). The single-cell RNA-seq results validate the expression of both the receptor isoforms (AdipoR1/R2) in retinal cells. The transcriptomic analysis showed lower expression of AdipoR1/2 in dry AMD pathogenesis compared to healthy subjects. The inhibitory adiponectin peptide (APN1) demonstrated over 75% suppression of CNV, whereas the control peptide did not exert any inhibitory effect on choroidal neovascularization (CNV). The elucidation of these relationships fosters a deeper understanding of adipose tissue’s profound influence on ocular health, presenting new prospects for personalized treatments and preventative measures. Because APN1 inhibits CNV and leakage, it can be used to treat human AMD, although the possibility to treat human AMD is in the early stage and more clinical research is needed. In conclusion, this review provides a captivating journey into the enthralling world of APN, intertwining the realms of adipose biology and ophthalmology in aging.

## 1. Introduction

Adiponectin (APN), a hormone linked to obesity regulation weighing around 30 kDa, is produced by the ADIPOQ gene and primarily originates from white adipose tissue. APN plays a crucial central role in various vital bodily functions such as managing glucose and fatty acid metabolism [1,2]. It also contributes to maintaining glucose and lipid balance [3], overall energy regulation [4], immune responses [5], and the effects of aging and metabolism [6,7,8]. Notably, APN extends its safeguarding influence on ocular tissue [8,9]. The retina, a metabolically very active component often outpacing even the brain’s metabolic rate, triggers blood vessel growth and regression due to its high-energy needs. APN has also demonstrated protective effects against multiple retinal disorders, including diabetic retinopathy (DR) [9], choroidal neovascularization (CNV) arising from age-related macular degeneration (AMD) [10], and other additional retinal complications [11].

Neovascular AMD is a complex retinal condition where an individual’s genetic disposition is influenced by the effects of age and environmental stressors. These factors cascade in a series of signaling pathways that involve inflammation, oxidation, and/or angiogenesis within the retinal pigment epithelial (RPE) cells and choroidal endothelial cells (CECs) [8]. Ultimately, this process results in vision loss due to the advancement of CNV. Navigating the world of AMD, we encounter its dual personas: the wet and dry types. Unveiling its dramatic impact, the wet form takes the spotlight, emerging as a chief instigator of irreversible blindness and the complete eclipse of central vision among the elderly. The hallmark of this drama is CNV [8,10]. In contrast, the dry type, though not synonymous with total blindness, casts a shadow over central vision, posing challenges in reading, driving, and perceiving the world around. As the curtain rises on advanced stages, dry AMD can take a perilous turn, progressing into geographic atrophy (GA) or even evolving into its wet counterpart, both orchestrating a symphony of severe vision loss [10]. Initiating a cascade of events, retinal hypoxia triggers an upsurge in metabolic demands, setting off signaling pathways that endeavor to tap into new vascular resources, ultimately culminating in the eye’s neovascularization [10]. The literature hints at a shift in the balance of two predominant circulating adipokines, APN and leptin, pivotal players in metabolic modulation across diverse tissues. This dynamic duo might play a role in driving the progression of neovascular eye conditions [9]. Further insights point to the heightened release of leptin, a hormone originating from adipocytes, as a harbinger of disrupted energy equilibrium, increased oxidative stress on vascular endothelial cells (ECs), and consequent dysfunction of these cells, ultimately contributing to retinopathy [12]. In parallel, another metabolic influencer, primarily sourced from adipocytes, APN, joins the orchestra of metabolic irregularities in the retina. The levels of circulating APN are intricately tied to DR [13,14], the development and advancement of premature retinopathy [15], and age-related macular degeneration [16]. This correlation is underscored by research, including studies involving laser-induced choroidal neovascularization [17,18] and a rodent model of oxygen-induced proliferative retinopathy, where higher circulating APN levels correlate with suppression of pathological vascular proliferation [19]. Embarking on a journey through ongoing investigations and pertinent studies, we delve into this burgeoning realm of APN/AdipoRs, dissecting their roles in the intricate landscape of retinal neovascular disorders.

## 2. Unveiling Adiponectin/AdipoRs Physiological Roles

### 2.1. Unlocking the Mysteries of APN/AdipoRs: A Journey through Discovery, Structure, and Forms in Circulation

During the mid-1990s, APN’s discovery unfolded as a harmonized effort across four research laboratories [20,21,22,23]. In contrast, the identification of APN receptors denoted as AdipoRs, underwent a protracted gestation period, finally coming to fruition in 2003 through pioneering work by Yamauchi et al. [24]. This milestone was achieved by selectively extracting two strongly associated seven-transmembrane receptors isoform, AdipoR1 and AdipoR2, from human skeletal muscle [25]. The foundational architecture of APN is constructed with a carboxy (C)-terminal globular domain paired with an amino (N)-terminal collagen-like domain [26]. The AdipoRs adopt the form of integral membrane proteins, where the N-terminus faces internally, and the C-terminus faces externally—a distinctive arrangement that diverges from the topology and role of other recognized G protein-coupled receptors (GPCRs) [27]. In a pattern of ubiquity, APN and AdipoRs manifest their presence across diverse tissues [28]. In addition to the dynamic duo of AdipoRs, APN extends its influence through interaction with the receptor T-cadherin, although, at present, its role seems less pivotal in comparison to AdipoRs [29].

The obesity-related peptide APN assumes a complex structural configuration and circulates within the bloodstream in different molecular forms: a trimer, hexamer, and a higher molecular weight (HMW) oligomer. These diverse APN variants exhibit varying levels of biological activity, with HMW oligomer APN being identified as the biologically energetic iteration of this hormone [30]. Interestingly, in specific scenarios, the HMW form has demonstrated superior insulin-sensitizing properties when compared to trimers or hexamer forms. In addition to its intricate structural diversity, APN undergoes glycosylation, a crucial post-translational modification necessary for maintaining its functionality. Its concentration in circulation typically ranges from 3 to 30 µg/mL in both humans and rodents, making it one of the most abundant adipokines present in the plasma [31,32].

### 2.2. Tissue Distribution, Mechanism, Physiological and Pathological Relevance of APN/AdipoRs Pathway

Primarily originating from adipocytes, APN is expressed in various locations in addition to plasma and imparts favorable influences on several metabolically demanding organs and cell types [33,34]. These include liver parenchymal cells (PCs), such as hepatocytes [35], skeletal muscle and myocytes [36], the brain [37], blood vessels [38], and reproductive organs in both males and females [6,39], as well as ocular tissues [9,40]. AdipoR1 and AdipoR2 exhibit broad and abundant expression, not limited to skeletal muscle and liver tissues but also extending to macrophages [41], the hypothalamus [42], white adipose tissue [43], reproductive tissues [44,45], and the retina [46]. In both in vitro and in vivo studies, AdipoRs have emerged as pivotal mediators of APN signaling [8]. APN engages with its two well-established, distinct cell-surface receptor variants, AdipoR1 and AdipoR2 [24]. Furthermore, AdipoR1 plays a more prominent role in initiating the AMP-activated protein kinase (AMPK) pathway, leading to the inhibition of hepatic glucose production and an increase in fatty acid oxidation. On the other hand, AdipoR2 is primarily associated with activating the peroxisome proliferator-activated receptor alpha (PPARα) nuclear receptor pathways, which in turn promote fatty acid oxidation and mitigate tissue inflammation and oxidative stress [47]. In metabolic organs targeted by insulin, such as the liver and skeletal muscle, the expression of AdipoRs significantly increases during fasting conditions compared to refed conditions in rodent models. Additionally, in vitro studies have revealed that insulin reduces the expression of AdipoRs through the phosphoinositide 3-kinase/FoxO1-dependent pathway [48]. The levels of APN in circulation and the presence of AdipoR1/R2 expression in metabolically active organs remain lower in obese and diabetic individuals as compared to healthy and lean individuals [49,50,51]. There is a significant reduction in APN concentrations among obese patients with Type 2 diabetes (T2D) and infertility [52]. In most cases, insulin acts as a stimulating factor, while tumor necrosis factor-alpha (TNF-α) serves as an inhibitor of APN signaling and secretion [53]. Additionally, it exerts a controlled influence on inflammatory responses by mitigating the production and functional activity of tumor necrosis factor-alpha (TNF-α) and interleukin-6 (IL-6) within macrophages through the inhibition of NF-κB activation, as elaborated earlier [54]. Furthermore, APN is recognized for its specific actions in regulating metabolism and developing insulin sensitivity.

APN also plays a vital role in managing multiple physiological processes, including glucose utilization, lipid biosynthesis, energy homeostasis, and inflammatory and retinal function [15,55]. Lack of APN secretion and expression globally or locally leads to insulin resistance, glucose intolerance, and hyperlipidemia in rodents [56,57]. APN along with other adipocytokines plays a primary pathophysiological function in the interaction between metabolism and reproduction and may be associated with the detrimental effect of aging on male reproductive actions [7,39]. The use of APN supplementation could potentially serve as a crucial therapeutic approach for addressing reproductive disorders associated with obesity, such as male and female infertility [6,58]. Knockdown experiments involving the ADIPOQ gene in skeletal muscles demonstrate the pivotal function of AdipoR1 in orchestrating various processes, including but not limited to β-oxidation, AMPK and PPAR pathway activation, glucose uptake [59]. AdipoR1 plays a role in elevating the phosphorylation of AMPK within the liver, consequently affecting the gluconeogenesis process. Concurrently, AdipoR2 is accountable for the removal of reactive oxygen species (ROS) and the initiation of nuclear receptor PPAR activation, along with the downstream modulation of target genes associated with β-oxidation. The emergence of nonalcoholic fatty liver disease (NAFLD) and steatohepatitis (NASH) accompanied by fibrosis and inflammation in obese rats fed a high-fat/high-cholesterol diet highlights the involvement of AdipoRs in regulating hepatic fatty acid metabolism. The decreased expression of AdipoRs isoforms during NASH was linked to lower levels of PPARα and AMPKα 1/2. Furthermore, specific tissues played a crucial role in determining these effects. AdipoR1 in the liver played a role in activating AMPK, while AdipoR2 was actively engaged in activating PPARα, resulting in heightened insulin sensitivity [60,61]. Due to their proficiency in establishing cross-organ communication and its lipid sequestration capabilities, APNs assumes an essential role in the preservation of lipid and glucose homeostasis. In a particular investigation, the overexpression of adiponectin and its associated receptors (AdipoRs) yielded numerous favorable outcomes, notably the reduction of visceral adiposity, amelioration of inflammatory responses, and mitigation of hepatic fibrosis [62].

## 3. Unlocking the Potential of APN/AdipoRs as Metabolic Regulators in Retinal Diseases

Recent investigations have explored the existence of APN/AdipoRs in ocular tissues [46,63], with a particular focus on its role in conditions such as DR, retinopathy of prematurity, the preservation of hypoxia-induced retinal neovascularization, photoreceptor integrity, retinitis pigmentosa, and AMD within the context of ocular pathophysiology. As previously discussed, it is important to note that the retina is among the most metabolically demanding tissues in the human body, and photoreceptors, in particular, house a greater number of mitochondria even relative to cardiomyocytes [64]. The retina is supplied with essential nutrients and oxygen through its vascular network. Premature loss of these blood vessels can induce hypoxia and insufficiency of energy substrates, both of which are recognized as pivotal factors in instigating angiogenesis within retinal tissue. Hypoxia leads to a reduction in the activity of prolyl hydroxylase, an enzyme well known for its capacity to rapidly degrade the hypoxia-inducible factor (HIF)-1 protein under normal oxygen conditions. Elevated levels of HIF-1 protein, in turn, initiate the expression of angiogenic factors, most notably vascular endothelial growth factor A (VEGFA) [65]. It is worth noting that specific metabolic pathways, independent of HIF-1, can also influence the regulation of VEGFA expression [65]. VEGFA plays a pivotal role in promoting the proliferation of blood vessels, a crucial response aimed at restoring oxygen and energy substrate supply to the retina. However, these newly formed blood vessels often exhibit structural abnormalities that may potentially damage the delicate retinal tissue [66], and in severe cases, this can progress to blindness.

Neurodegenerative eye diseases often manifest with symptoms such as hazy, blurred, or distorted vision. The activation of the APN/AdipoRs signaling pathway has demonstrated significant neuroprotective potential, offering promise for ameliorating these conditions and enhancing visual function. Notably, both APN and AdipoRs are found within various retinal cells. When APN binds to AdipoRs and subsequently triggers downstream molecular pathways, it exerts its therapeutic effects, with detectable expression in the retina. While adipose tissues predominantly secrete APN [43], it is worth mentioning that the retina [46] and brain [37] can also locally produce this protein. Furthermore, APN readily traverses the bloodstream and efficiently crosses the blood–brain barrier. Numerous pathophysiological disease states, such as elevated blood glucose levels, mitochondrial dysfunctions and dyslipidemia, have the potential to disrupt retinal functions that play a major role in the development of retinal vascular complications [67]. A pivotal driver of glycolysis, specifically isoform 3 of 6-Phosphofructo-2-kinase/fructose-2,6-bisphosphatase, plays a vital role in the regulation of blood vessel formation [68]. Perturbations in the activity of glucose metabolic enzymes within the polyol pathway can also assume a protective role for the retina, safeguarding it against retinal dysfunction and abnormal blood vessel growth [69,70]. Furthermore, an alternative approach to curbing vessel sprouting involves inhibiting the rate-limiting fatty acid oxidation enzyme, namely, carnitine palmitoyl transferase 1 [71].

Several studies have explored the protective function of APN/AdipoRs pathways, and one noteworthy investigation was conducted among individuals diagnosed with T2D in Japan. Utilizing laser Doppler velocimetry, the study observed that in males, there was a positive correlation between blood APN levels and retinal blood vessel diameter, as well as retinal blood velocity and flow [72]. However, such a correlation was not observed in females. Achieving a better balance in one’s lifestyle or utilizing medications that lead to an elevation in plasma APN levels may unveil a promising avenue for the development of innovative therapeutic strategies in diabetes treatment [72]. It is worth noting that high glucose levels have been identified as the primary predisposing factor for angiogenesis in DR. Findings from a previous study have elucidated the role of APN in the dysregulated autophagy process and retinal angiogenesis [73]. Additionally, APN has exhibited a protective effect against high glucose-induced damage to RF/6A cells. Furthermore, it has been shown to mitigate high glucose-induced angiogenesis in chorioretinal endothelial RF/6A cells by inhibiting the autophagy pathway [73]. This research indicates that APN is a promising therapeutic target for treating angiogenesis in diabetic retinopathy (DR). It can effectively reduce retinal neovascularization by inhibiting tube formation in human cell cultures of retinal microvascular ECs, umbilical vein macrovascular ECs, and choroidal ECs. This effect is associated with the suppression of VEGF’s role in DR-related angiogenesis [74,75]. The closely related cytokine, C1q/TNF-related protein-9, can help maintain the blood-retinal barrier (BRB), reducing inflammation in diabetic db/db mice with DR [40].

## 4. Current Understanding of the Pathophysiological Role of APN/AdipoRs in Neovascular AMD

Neovascular AMD is one of the major factors in allowed blindness in the elderly. This progressive disease affects the macular region (also called macula lutea) of the eye, a pigmented yellow area mass of the retina, containing color-sensitive rods, which is vital for sharp, central vision. Evidence from the literature suggests a crucial role of APN in ameliorating neovascularization in AMD [8,17]. Mice with laser-induced neovascularization in the choroid serve as a model to simulate various inflammatory responses associated with AMD [17,18]. The growing body of research indicates that Adiponectin (APN) may emerge as a novel and highly promising therapeutic target for addressing angiogenesis associated with DR. More specifically, it has the potential to significantly reduce the development of new blood vessels in the retina, a process known as retinal neovascularization, in primary human cell cultures of retinal microvascular endothelial cells (ECs), umbilical vein macrovascular ECs, and choroidal ECs. This beneficial effect is closely linked to its ability to interfere with the function of the VEGF, a key driver of angiogenesis in DR.

Importantly, the most closely related counterpart of APN, a cytokine called C1q/TNF-related protein-9, has shown the capability to protect the integrity of the blood–retinal barrier (BRB). Preserving the BRB serves to reduce the inflammatory response observed in diabetic db/db mice affected by DR. In addition to its potential applications in treating DR, therapies centered around APN/AdipoRs may hold promise in addressing the vision-threatening consequences of AMD. AMD encompasses two prevalent phenotypes: dry AMD and wet AMD. This condition is considered by the accumulation of drusen, which are composed of a mixture of proteins, fats, minerals, and other debris, forming spherical structures bound tightly to proteins. With age, drusen inflict damage on the retina, leading to permanent changes in retinal cells. Dry form can progress to wet form, described by the development of new blood vessels originating from the choroid. These vessels grow in the subretinal space including RPE and, ultimately culminating in central significant vision loss. Mallordo and colleagues have postulated the distinct role of APN in ocular diseases, highlighting its inhibitory effects on the proliferation and migration of RPE cells [76]. Furthermore, Osada and his team demonstrated the consequences of AdipoR1 deletion on abnormal lipid metabolism within the retina, as well as retinal neurodegeneration, using AdipoR1 deleted mice model. Their research, utilizing in situ hybridization, revealed robust AdipoRs mRNA expression in the photoreceptor inner segment (PIS) and faint reactivity in the inner retinal layers in 4-week-old control mouse retinas. The expression of AdipoR1 in the retina appears to play a crucial role in inducing the elongase enzyme of very long-chain fatty acids (ELOVL2), a possibly essential step in providing an adequate supply of docosahexaenoic acid (DHA) necessary for the proper functioning and survival of photoreceptor cells [77].

**Figure 1:**
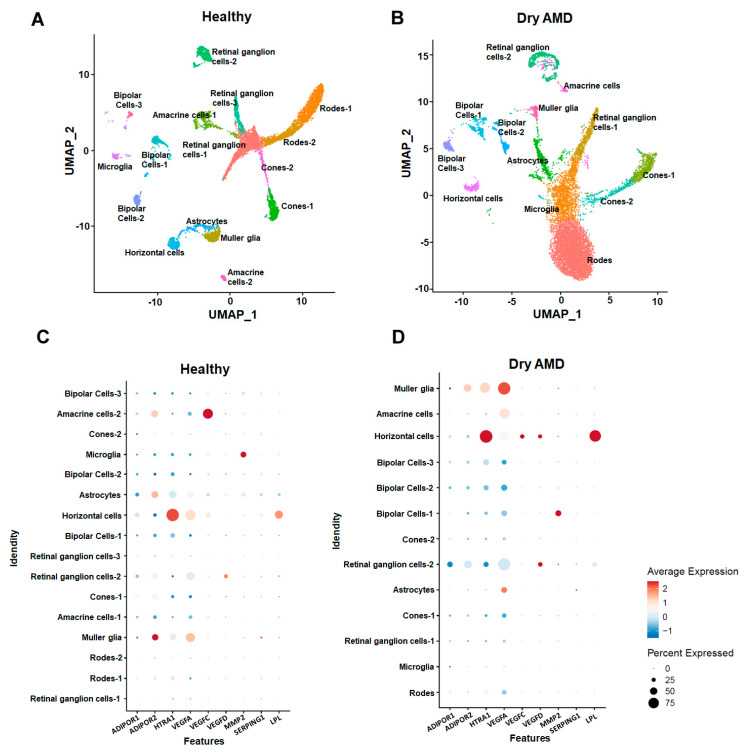
Single-cell UMAP visualization depicting the cellular landscape of healthy (**A**) and dry age-related macular degenerated (AMD) eyes (**B**). The expression of AdipoR1 and AdipoR2 genes in various retinal cell populations of healthy (**C**) and diseased dry AMD (**D**). Data were analyzed using the software Seurat v4.1.1 implemented in R v4.2.1.

Potentially, the expression of APN and its receptors in single-cell RNA-seq datasets related to AMD can help researchers better understand the possible mechanism of APN/AdipoRs signaling in retinal disease, including its impact on inflammation, angiogenesis, and specific retinal cell populations. This knowledge may ultimately contribute to the development of more effective treatments for AMD. To date, none of the literature on single-cell RNA-seq presented the expression of APN/AdipoRs in the various retinal cell populations, which emphasizes the need to enumerate the APN pathway genes during AMD pathogenesis. The single-cell RNA-sequencing data for dry AMD were obtained from the GEO database (https://www.ncbi.nlm.nih.gov/geo (accessed on 15 August 2023)) under accession number GSE221042. We conducted an in-depth analysis of cellular heterogeneity and landscape using the Seurat package v4.1.1 [78] by plotting UMAP (Uniform Manifold Approximation and Projection). Furthermore, we identified distinct cell types using the corresponding marker genes (Figure 1A,B) as previously reported by Kuchroo et al. [79]. By employing marker genes, we were able to distinguish various types of neuronal cells, such as retinal ganglion cells, horizontal cells, bipolar cells, rod photoreceptors, cone photoreceptors, and amacrine cells, in addition to uncovering infrequent non-neuronal cell types, such as microglia, astrocytes, and Müller glia. To understand the cellular expression of APN/AdipoRs and how the AdipoR1/R2 changes during AMD pathogenesis, we plotted feature dot plots (Figure 1C,D). Our results demonstrate that expression of both the receptor isoforms (AdipoR1/R2) showed lower expression in dry AMD pathogenesis compared to healthy subjects. Furthermore, we checked the expression of other vascular cell subpopulations. Our analysis revealed the elevation of angiogenesis marker genes VEGFA in Müller glia cells and AMD-associated genes HTRA1 in the horizontal cells during dry AMD pathogenesis. Dry AMD is characterized by the activation of intrinsic immune cells within the retina, specifically microglia cells, Müller cells, retinal pigment epithelial (RPE) cells, and macrophages. Under dry AMD pathophysiological conditions, Muller cells are involved in retinal angiogenesis [80]. Notably, drusen, a hallmark feature of dry AMD, contains a plethora of pro-inflammatory proteins, including apoE protein, acute phase and coagulation proteins, immunoglobulin G (IgG), complement components, and activators [81]. This indicates that local inflammatory response (ocular) plays an essential role in the early pathophysiology of AMD.

Bushra and colleagues noted that APN played an inhibitory role in the adhesion of endothelial cells (ECs) and the organization of the extracellular matrix. Simultaneously, this led to an enhancement in the barrier function, effectively mitigating the damage induced by high glucose levels in human retinal endothelial cells (HMRECs) [82]. In another study using a diabetic mouse model induced by STZ, researchers examined the impact of APN on the early development of vascular system damage in the retina. The immunofluorescence findings revealed that APN localized in the vascular endothelium of retinal arterioles in a T-cadherin-dependent manner, which progressively declined as diabetes progressed. This decline in retinal APN expression was concurrent with early signs of DR, characterized by increased vessel permeability. Importantly, treatment with dapagliflozin, a selective inhibitor of sodium–glucose co-transporter 2 aimed at lowering glucose levels, effectively prevented this reduction in retinal APN/AdipoRs system expression [83]. Furthermore, the study found that a deficiency in APN resulted in pronounced vascular permeability during moderately short-term hyperglycemia. This was accompanied by a significant increase in vascular cellular adhesion molecule-1 (VCAM-1) and a decrease in claudin-5 expression in the endothelium region of the retina [83,84].

**Figure 2:**
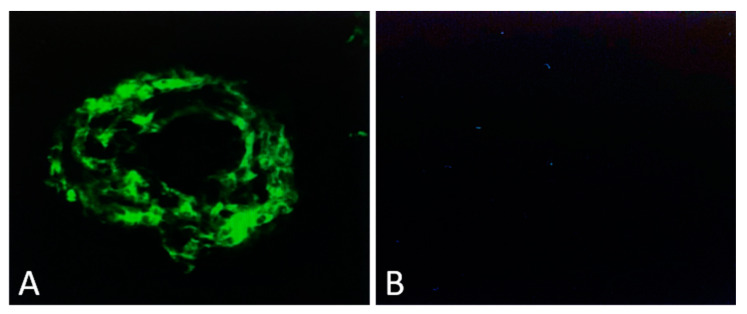
Laser-induced CNV in mice. Four laser spots were placed in each mouse eye. Animals were sacrificed on day 7 (CNV fully matures on day seven in this model), and confocal pictures were taken after making flat mounts. The green color indicates new vessels formed from choroid (**A**). Phospho-buffer saline (PBS)-treated mice did not show any green color, indicating no CNV formation from the choroid (**B**).

Mice deficient in very low-density lipoprotein receptors (Vldlr KO) exhibit pathological angiomatous proliferation in the retina, a disease state that also afflicts people with AMD. A supplementary ingredient ω3 long-chain polyunsaturated fatty-acid (ω3-LCPUFA), added to rodent food in the form of docosahexaenoic acid (DHA) and eicosapentaenoic acid (EPA), is identified to suppress laser-induced CNV in controlled mice whereas this suppression is abolished in APN deficient mice. In addition, in the retinas of Vldlr KO mice, the ω3-LCPUFA can further enhance its receptor1 expression and inhibit CNV. The clinical and experimental evidence suggests that ω3-LCPUFA-rich food may serve a protective role for AMD patients. In another investigation employing a choroid mice model with laser-induced neovascularization, it was demonstrated that the magnitude of choroidal neovascularization (CNV) could be significantly decreased through the administration of Adiponectin Peptide I (APN1) [15,16,85]. Adiponectin (APN) is recognized for its anti-inflammatory properties and shares over 75% structural similarity with the complement protein C1q. APN1 peptide, derived from the globular domain of APN, exhibited a remarkable inhibition of CNV, surpassing 75% reduction when administered subretinally in comparison to the control peptide [17,18]. CNV was analyzed by confocal microscopy by measuring newly formed green vessels. Newly formed vessels were stained green by perfusion of FITC-Dextran. Image analysis was performed using the ImageJ program (Figure 2).

**Figure 3:**
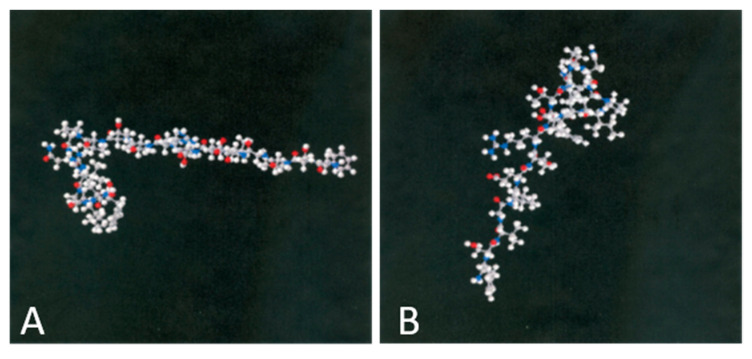
Three-dimensional (3D) structure of Control peptide. (**A**) FSVGLETRVTVPNVPIRF and APN1 inhibitory peptide (**B**) KDKAVLFTYDQYQEKNVD. All modeling calculations were performed using the SYBYL program, and the structure of the peptide was built using the ‘build protein’ tool in SYBYL. The lowest energy conformer for the peptide was calculated to be 43.145 hartrees [17].

We have designed and synthesized several peptides. APN1, APN2, APN3 and Control peptide. After scanning all the peptides, APN1 inhibited CNV by more than 75%. APN2 and APN3 inhibited CNV by 50% and 40%, respectively. The control peptides did not inhibit CNV at all. Therefore, we used APN1 and Control peptide for our experiments (Figure 3). All control peptides were designed with the same number of amino acids as APN1. We think APN1 may be a better alternative for the most frequently used wet AMD treatments. Anti-VEGF treatments can cause hemorrhage, require frequent injections, and are very expensive for patients. APN1 is not an anti-VEGF treatment. Rather, it binds to Adiponectin receptor one (AdipoR1), then, through cAMP, it inhibits CNV development [86,87].

The wet form of AMD is characterized by an excessive growth of new blood vessels around the macula, a region of the retina. This abnormal process is called CNV, which can lead to the development of leaky blood vessels. Therefore, the vision loss associated with wet AMD tends to be more severe compared to the dry form. Recent research indicates that a peptide known as Adiponectin Peptide 1 (APN1) has demonstrated the ability to decelerate the advancement of CNV. We have designed a peptide APN1 as an agonist and we think, for the purpose of continuous APN1 activity, it can be pegylated for clinically proven effect. Various doses of APN1 can be evaluated in the rodent model to offer clinical insights regarding the optimal dosing for potential human applications. The best dose was 20 μg/kg, and this will give us some idea about the dose to be used in humans. Once we know the binding mechanism of APN1/AdipoRs, by our experiments or from a literature search, we can design an inhibitor and test it to proceed further to find out whether this inhibitor will also block APN1 binding. Its well established where APN binds to AdipoR1 but not the peptide APN1 that we have designed [28,55,60]. Currently, we are unsure how well APN1 will inhibit wet AMD in humans, but we expect it will be less invasive as an eye drop or may require fewer injections with greater intervals between treatment visits compared to currently available drugs on the market today.

## 5. Exercise, APN/AdipoRs Signaling, and Neovascular AMD

Physical exercise has the potential to excite the systemic and localized production of APN, offering a protective effect in various ocular conditions, including but not limited to DR, AMD, retinitis pigmentosa (RP), glaucoma, and light-induced retinal degeneration [88]. Engaging in daily physical exercise seems to provide a safeguard against the development of neovascular AMD. Recent studies observed that AIM2/NLRP2 inflammasome (predominantly expressed in microglia) was most copiously expressed in the retina–choroid complex. They found that the expression of AIM2 was significantly induced during CNV pathogenesis which was significantly attenuated by treadmill training exercise in rodents [88]. Physical exercise mitigates neovascular AMD by suppressing the AIM2 inflammasome in myeloid cells. Moreover, earlier proteomic data directed that physical exercise facilitated the secretion of APN [9], from fat to circulation, which in turn, led to a decrease in ROS-induced DNA damage and the inhibition of AIM2 inflammasome activation in myeloid cells within eyes affected by choroidal neovascularization (CNV), and this effect was facilitated through the AMPK-p47phox pathway. Findings from earlier studies suggested that in sedentary AdipoQ-deficient (APNKO) mice, there was a tendency for an enhanced leakage area and choroidal neovascularization (CNV) volume when compared to sedentary wild-type mice [88]. Furthermore, this difference became more pronounced in exercised mice, implying that APN plays an essential role in the positive effects observed in exercised mice [89]. Earlier investigation aimed to ascertain if exercise induced an enrichment of APN within the ocular structures. Utilizing Western blotting and ELISA assays, they demonstrated a noteworthy elevation in APN levels within the retina–choroid complex and the vitreous fluid of the exercised mice [90]. Notably, while treadmill exercise training significantly reduced the leakage area and CNV volume in wild-type mice, this protective effect was not statistically significant in APNKO mice [88]. These results substantiate the essential role of APN/AdipoRs in mediating the protective benefits of exercise. To obtain more comprehensive results, it is imperative to implement more detailed and rigorous research studies.

## 6. Adiponectin’s Antioxidative Properties in Ocular Tissues

Oxidative stress assumes a pivotal role in the pathogenesis of age-associated ocular diseases, notably age-related macular degeneration (AMD), cataracts, and glaucoma. Advancing age is associated with a declining capacity for antioxidant defense mechanisms, leading to an accumulation of reactive oxygen species (ROS) within diverse ocular cell types. This surge in ROS levels precipitates oxidative damage, a hallmark of age-related ocular pathologies [91]. APN hormone has garnered increasing attention for its anti-oxidative properties within the ocular tissues. Emerging research has demonstrated that APN exerts a multifaceted anti-oxidative influence on retinal structures, particularly in the context of aging-related ocular diseases such as AMD and DR. APN appears to modulate oxidative stress by downregulating ROS generation, mitigating lipid peroxidation, and bolstering antioxidant defense mechanisms. Additionally, APN’s capacity to enhance EC function and mitigate inflammation plays a pivotal role in its anti-oxidative effects, as inflammation and oxidative stress are often intertwined in ocular pathologies [92]. The intricate interplay between APN and oxidative stress pathways in retinal cells presents a promising avenue for future investigations and therapeutic interventions aimed at preserving visual health.

## 7. Unlocking Clarity: Adiponectin’s Transformative Quest from Fat Reserves to Optical Resilience

The multifaceted obesity hormone APN has evolved from being initially perceived as a critical regulator of adipose tissue metabolism to a multifunctional molecule with far-reaching effects across various physiological domains. Beyond its contributions to glucose homeostasis, lipid metabolism, and insulin sensitivity, recent research has unveiled APN’s involvement in unexpected domains, such as optical resilience. Intriguingly, APN appear to exert a protective influence on vision, especially retinal function, a feat that illuminates its versatile nature. This newfound role is rooted in its ability to modulate inflammatory responses, maintain vascular integrity, and mitigate oxidative stress within the intricate microenvironment of the eye. By suppressing the production of inflammatory cytokines, such as TNF-α and interleukin-6 (IL-6), APN helps to create a less hostile environment for retinal cells [83]. Moreover, its vasodilatory properties contribute to improved blood flow, which is crucial for maintaining the highly vascularized retina’s health. Additionally, APN’s antioxidant effects further shield the delicate retinal structures from oxidative damage. This newfound role in safeguarding the delicate structures of the eye underscores the profound and far-reaching impact of APN on overall health. As we delve deeper into the mechanisms underpinning its actions, the journey of APN from adipose reserves to retinal resilience continues to illuminate novel avenues for therapeutic interventions and a more comprehensive understanding of the intricate interplay between metabolic and physiological processes.

## 8. Future Directions for Research and Clinical Applications

The multifaceted benefits of APN/AdipoRs signaling have been demonstrated across various cell types, encompassing insulin-sensitizing actions, anti-inflammatory effects, anti-atherosclerotic properties, anticarcinogenic potential, and antiproliferative activities. Given APN’s ameliorative role in mitigating insulin resistance, diabetes, and age-related conditions, a decline in APN levels is deemed pivotal in the pathophysiology of retinal diseases. It is also associated with the susceptibility to diabetes-related diabetic retinopathy (DR) and age-related macular degeneration (AMD)-related neovascularization. The investigation of the critical roles of AdipoR1 and AdipoR2 has gained momentum following the cloning of these two adiponectin receptors, which confirmed their essential role in APN binding and its subsequent glucose-lowering effects. Moreover, APN activation can trigger the activation of AMPK/SIRT1/PGC-1α and nuclear receptors PPARs through the AdipoRs signaling pathway. The screening of low molecular weight compounds to identify AdipoR1/R2 agonists, along with various therapeutic approaches, offers the potential to develop novel therapeutic strategies. Utilizing 3D conformational analysis of AdipoRs, optimization of AdipoRs agonists can be achieved, enabling the development of effective, safe, and high-quality drugs for treating debilitating eye conditions. Future research endeavors should prioritize elucidating the functions of AdipoR1 and AdipoR2 and targeting their agonists to create innovative anti-aging and anti-diabetic pharmaceuticals. This approach not only enhances our understanding of the molecular mechanisms underlying APN’s activity but also contributes to addressing obesity-related and other metabolic disorders.

Moreover, age-related macular degeneration (AMD), diabetic retinopathy (DR), and retinopathy of prematurity have all been linked to disruptions in the circulating function of APN/AdipoRs or alterations in the distribution of APN variants. In experimental settings, APN has shown its potential to counteract retinal defects and choroidal neovascularization (CNV). Given its pivotal role as a regulator of glucose and lipid metabolism, APN-derived peptides hold promise in restoring metabolic equilibrium. Interventions involving ω3 long-chain polyunsaturated fatty acids (LCPUFA) and fibric acid derivatives have been shown to boost APN levels in the bloodstream. Physical exercise has the capacity to stimulate systemic and local APN production, contributing to its protective effects in various ocular diseases, including DR, AMD, retinitis pigmentosa (RP), glaucoma, and light-induced retinal degeneration. Further planned investigations are necessary to deepen our understanding and elucidate the role of APN/AdipoRs in neovascular AMD. Additionally, research efforts should aim to uncover the underlying molecular mechanisms, thus advancing our comprehension of both the experimental and clinical implications of this pathway.

## 9. Conclusions

In conclusion, “From Fat to Sight” provides a captivating journey into the enthralling world of APN/AdipoR1/2, intertwining the realms of adipose biology and ophthalmology. This innovative review not only piques the curiosity of researchers but also holds promise in revolutionizing future approaches to combating aging-associated eye disorders. In this comprehensive review, we aim to provide a deeper understanding of the intricate relationship between adiponectin and eye disorders associated with aging. By elucidating the molecular mechanisms, clinical associations, and therapeutic implications, we hope to inspire further research and innovative strategies for the prevention, diagnosis, and treatment of ocular diseases. The remarkable connection between fat, in the form of adiponectin, and sight opens exciting avenues for advancing eye health and improving patient outcomes. Our future plans are to conduct clinical studies using APN1 and control peptides, injecting both in human vitreous separately to see if this peptide [APN1] can be used for the treatment of human AMD compared to control peptide because we know that APN1 inhibits CNV and leakage in animal models.

## Data Availability

Not applicable.
